# Ninjin'yoeito in the Management of Frailty and Overactive Bladder in Elderly Women: A Report of Two Cases

**DOI:** 10.7759/cureus.69304

**Published:** 2024-09-13

**Authors:** Nobuo Okui

**Affiliations:** 1 Dentistry, Kanagawa Dental University, Kanagawa, JPN

**Keywords:** frailty, fried frailty criteria, kampo medicine, mini-mental state examination, ninjin'yoeito, overactive bladder, pittsburgh sleep quality index

## Abstract

This case report examines the efficacy of Ninjin'yoeito (NYT), a traditional Japanese Kampo medicine, in managing frailty and overactive bladder (OAB) in two elderly female patients. We present two cases: an 84-year-old woman and an 80-year-old woman, both experiencing decreased physical strength, OAB, insomnia, and anxiety. The 80-year-old patient also exhibited cognitive decline.

After three months of NYT treatment (7.5 g/day, Kracie Pharma Ltd., Tokyo, Japan), both patients showed significant improvements. The 84-year-old patient's frailty status improved from frail to pre-frail, with enhancements in grip strength (16 kg to 18 kg), urinary symptoms, and Short Form-36 scores. Her total testosterone levels increased from 14 ng/mL to 28 ng/mL. The 80-year-old patient demonstrated marked improvements in cognitive function (Mini-Mental State Examination score increased from 12 to 23), physical activity, and OAB symptoms. Her grip strength increased from 12 kg to 18 kg, and total testosterone levels rose from 20 ng/mL to 38 ng/mL.

Both patients experienced reductions in urinary frequency and nocturia. The 84-year-old patient's Overactive Bladder Symptom Score (OABSS) improved, particularly in urgency and urgency incontinence. The 80-year-old patient's OABSS showed significant improvements across all components. Sleep quality, assessed by the Pittsburgh Sleep Quality Index, improved for both patients.

These cases suggest that NYT may be an effective and well-tolerated treatment option for elderly patients with concurrent frailty and OAB, potentially addressing multiple geriatric issues simultaneously. The observed increases in testosterone levels may contribute to the overall improvements. Further investigation in larger clinical studies is warranted.

## Introduction

Frailty in the elderly population is a growing concern in geriatric medicine, often coexisting with urological conditions such as overactive bladder (OAB) and lower urinary tract symptoms [1-5]. The management of these conditions in frail patients presents unique challenges, particularly owing to the increased risk of adverse effects from conventional pharmacological treatments [6]. Traditional Japanese Kampo medicine, specifically Ninjin'yoeito (NYT), has shown promise in addressing both frailty and associated genitourinary symptoms [2]. Recent studies have demonstrated the efficacy of NYT in improving frailty scores and alleviating symptoms related to OAB and genitourinary syndrome of menopause [2,3]. However, literature on individual patient responses to NYT in the context of frailty and urological symptoms remains limited. In this report, we present two cases of elderly female patients with frailty and concurrent OAB symptoms, who were treated with NYT. These cases aim to illustrate the potential benefits of NYT in managing both frailty and urological symptoms and highlight the importance of individualized treatment approaches in geriatric care. A detailed examination of these cases aims to contribute to the growing body of evidence supporting the use of NYT in frailty patients with urological conditions.

## Case presentation

These two cases were initially presented between December 2022 and June 2023. Since then, both patients have been followed up at our hospital to the present day. During this period, these two consecutive cases were the only patients who were able to respond to all the extensive questionnaires shown in this report.

Case 1

An 84-year-old woman presented to our clinic in December 2022 with the chief complaints of decreased appetite and reduced physical strength over the past two years, OAB for four years, insomnia for the past 12 months, and anxiety for the past six months. Her medical history included hypertension for over 10 years, as well as hyperuricemia and reflux esophagitis, though the onset dates of these conditions are unclear. Current medications included amlodipine besylate 10 mg, bisoprolol 25 mg, febuxostat 10 mg, and lansoprazole 15 mg. She had no history of uterine or ovarian diseases, bladder stones, bladder tumors, or interstitial cystitis.

The patient reported a gradual decline in appetite over two years, resulting in a weight loss of 10 kg. She experienced increased urinary frequency (nine times/day), nocturia (three times/night), and urinary urgency (with each void). Two months before her initial consultation at our clinic, we noted an additional 1 kg weight loss. Despite previous *H. pylori* eradication therapy for reflux esophagitis by her former physician, her symptoms persisted. The patient reported a loss of taste enjoyment and decreased ability to climb stairs at home.

Prior to visiting our clinic, the patient had been diagnosed with OAB, with an Overactive Bladder Symptom Score (OABSS) of 12 [1,2]. Her previous physician had initiated treatment with fesoterodine fumarate 4 mg approximately one year ago, which reduced her OABSS to 9 but caused severe dizziness, tachycardia, and increased intraocular pressure. About six months ago, her medication was switched to vibegron 50 mg, which exacerbated her dizziness and insomnia.

After physical examination and laboratory tests at our clinic, treatment was initiated. Physical examination revealed a height of 146 cm, weight of 39 kg (BMI: 18.3 kg/m²), blood pressure of 102/59 mmHg, pulse of 91 bpm, and temperature of 35.8°C. ECG was normal. Laboratory findings showed a hemoglobin of 10.5 g/dL, WBC of 3,500 /µL, albumin of 3.2 g/dL, serum creatinine of 0.6 mg/dL, C-reactive protein (CRP) of 0.1 mg/dL, total cholesterol level of 150 mg/dL, fasting blood glucose level of 90 mg/dL, aspartate aminotransferase (AST) level of 10 U/L, alanine aminotransferase (ALT) level of 7 U/L, total bilirubin of 0.3 mg/dL, and blood urea nitrogen (BUN) of 7 mg/dL.

The patient's cognitive function was assessed using the Mini-Mental State Examination (MMSE), with a score of 28 out of 30 before NYT administration [7]. The patient was prescribed NYT for three months. The patients were administered 7.5 g/day of NYT granules (KB-108, Kracie Pharma Ltd., Tokyo, Japan). Following treatment, the patient’s physical function and strength improved significantly. The patient’s appetite gradually increased, leading to a weight gain of 1 kg. She reported an improved ability to climb stairs, and her grip strength increased from 16 to 18 kg. The patient's total testosterone level increased from 14 ng/mL to 28 ng/mL after NYT administration, indicating a significant improvement in hormonal balance.

After NYT administration, the patient's total MMSE score remained stable at 28, indicating that cognitive function was maintained throughout the treatment period. Health-related quality of life was assessed using the Short-Form 36-Item Health. Survey (SF-36) questionnaire. Table 1 shows the significant improvements across multiple SF-36 components [8]. Activities of Daily Living also improved, particularly moderate exercise capacity, stair climbing, walking distance, and the ability to carry loads.

**Table 1:** SF-36 component scores before and after NYT administration SF-36: Short-Form 36-Item Health Survey; NYT: Ninjin'yoeito. Scores range from 0 to 100, with higher scores indicating better health status in each domain. Scores below 50 are considered below average health status.

SF-36 Component	Before NYT	After NYT
Physical Functioning (PF)	10	50
Mental Health (MH)	40	50
Social Functioning (SF)	50	60
General Health (GH)	40	50

The patient's insomnia and mental health had improved. Sleep quality was assessed using the Pittsburgh Sleep Quality Index (PSQI) [9]. As shown in Table 2, significant improvements were observed across all PSQI components, with the Global PSQI Score decreasing from 15 to 8, indicating a substantial enhancement in overall sleep quality. anxiety decreased and she reported improved mental stability. Daytime sleepiness was reduced, which enhanced her quality of daily life.

**Table 2:** Pittsburgh sleep quality index (PSQI) scores before and after NYT administration NYT: Ninjin'yoeito Component scores range from 0 to 3, with the Global PSQI Score ranging from 0 to 21. Higher scores indicate poorer sleep quality. A Global PSQI Score > 5 indicates poor sleep quality.

Pittsburgh Sleep Quality Index Component	Before NYT	After NYT
Subjective Sleep Quality	2	1
Sleep Latency	3	2
Sleep Duration	2	1
Sleep Efficiency	3	2
Sleep Disturbances	2	1
Use of Sleep Medication	1	0
Daytime Dysfunction	2	1
Global PSQI Score	15	8

Frailty indicators were evaluated before and after NYT administration (Table 3). Based on the Fried Frailty Criteria, the patient's frailty status improved from frailty (meeting four out of five criteria: exhaustion, weakness, slowness, and reduced physical activity) to pre-frail (meeting only one criterion: slowness) after three months of NYT treatment [1-4,10].

**Table 3:** Frailty indicators before and after NYT administration NYT: Ninjin'yoeito Scores are 0 (absent) or 1 (present) for each criterion, except slowness, which ranges from 0 to 2. Frailty classification: 0: Robust, 1-2: Pre-frail, 3 or more: Frailty.

Frailty Indicator	Before NYT	After NYT
Unintentional Weight Loss?	0	0
Exhaustion?	1	0
Weakness?	1	0
Slowness?	2	1
Reduced Physical Activity?	1	0

OAB symptoms showed marked improvement, with daytime urinary frequency decreasing from nine to six times/day, and nocturia reduced from three to one to two times/night. The frequency and intensity of episodes of urinary urgency and urge incontinence also decreased. The OABSS showed improvements in urgency and urgency incontinence, both of which decreased from 4 to 3, as shown in Table 4.

**Table 4:** OABSS before and after NYT administration OABSS: Overactive Bladder Symptom Score; NYT: Ninjin'yoeitoScores for individual symptoms range as follows: Daytime Urinary Frequency (0-2), Nocturia (0-3), Sudden Strong Urge to Urinate (0-5), and Urgency Incontinence (0-5). Total OABSS ranges from 0 to 15. Severity classification: Mild (0-5), Moderate (6-11), Severe (12-15). Higher scores indicate more severe OAB symptoms.

OABSS	Before NYT	After NYT
Daytime Urinary Frequency	3	3
Nocturia	3	3
Sudden Strong Urge to Urinate	4	3
Urgency Incontinence	4	3

Case 2

An 80-year-old woman presented to our clinic in June 2023 with the chief complaint of OAB, which she had been experiencing for over three years but had worsened rapidly in the past one year. Over the past five years, she also reported decreased physical strength, cognitive decline, insomnia, and anxiety. Her medical history included hypertension and diabetes mellitus (DM) since around the age of 65. Current medications included amlodipine besylate 10 mg and sitagliptin 12.5 mg, which remained unchanged throughout the treatment period.

The patient reported a gradual decline in motivation and increased urinary frequency (12 times/day) and nocturia (five times/night) over the past five years. Prior to the initial consultation at our clinic, her walking ability had deteriorated and she had stopped going out. Before visiting our clinic, she had been diagnosed with OAB, with an OABSS score of 13. Her previous physician had initiated treatment with fesoterodine fumarate 4 mg, which did not improve her OABSS and exacerbated her insomnia. Concerned about her heart health, she was hesitant to continue when the addition of vibegron 50 mg caused palpitations.

After physical examination and laboratory tests at our clinic, treatment was initiated. Physical examination revealed a height of 150 cm and a weight of 62 kg (BMI: 27.6 kg/m²). Laboratory findings showed hemoglobin of 11.5 g/dL, WBC of 4,000/µL, albumin of 3.5 g/dL, serum creatinine of 0.6 mg/dL, CRP of 0.1 mg/dL, total cholesterol of 150 mg/dL, fasting blood glucose of 80 mg/dL, AST of 15 U/L, ALT of 10 U/L, total bilirubin of 0.3 mg/dL, BUN of 8 mg/dL, and glycated hemoglobin (HbA1c) of 6.8%. All of these values were within the normal range, albeit showing a slightly low trend.

Fesoterodine fumarate 4 mg was discontinued and NYT treatment was initiated simultaneously. The patient was prescribed NYT for three months. Following treatment, her physical function and strength improved significantly. She reported being able to take a 45-minute walk daily and climb stairs more easily. Her grip strength increased from 12 kg to 18 kg. Notably, the patient's total testosterone level increased from 20 ng/mL to 38 ng/mL after NYT administration, indicating a substantial improvement in hormonal balance.

The patient's cognitive function was assessed using the MMSE before and after NYT administration [11]. Table 5 shows the changes in MMSE scores across the different cognitive domains. MMSE results demonstrated significant improvements across all cognitive domains following NYT treatment. The total MMSE score increased from 12 to 23, indicating substantial enhancement in overall cognitive function. Notably, the patient showed marked improvements in orientation, attention, calculation, and language skills.

**Table 5:** MMSE scores before and after NYT administration MMSE: Mini-Mental State Examination; NYT: Ninjin'yoeito. Total MMSE score ranges from 0 to 30, with higher scores indicating better cognitive function. Each component has a different maximum score: Orientation (10), Registration (3), Attention and Calculation (5), Recall (3), Language (8), and Visuospatial Ability (1). 24-30: No cognitive impairment, 19-23: Mild cognitive impairment, 10-18: Moderate cognitive impairment, ≤9: Severe cognitive impairment.

MMSE Component	Before Treatment	After Treatment
Orientation	3	6
Registration	2	3
Attention and Calculation	1	3
Recall	0	2
Language	3	5
Visuospatial Ability	3	4

After NYT treatment, substantial improvements were observed across all measured domains of the SF-36 questionnaire. Physical Functioning increased from 10 to 50, Mental Health from 40 to 60, Social Functioning from 35 to 55, and General Health perception from 35 to 60. These improvements aligned with the patient's reported enhancements in daily activities, mental state, and overall well-being.

Modest improvements were observed in several PSQI components after the treatment. The Global PSQI Score decreased from 29 to 24, indicating a slight enhancement in the overall sleep quality. Subjective Sleep Quality, Sleep Latency, Sleep Duration, and Sleep Efficiency improved marginally, with scores decreasing from five to four in each category. Sleep disturbances and the use of sleep medications remained unchanged at 4. Daytime Dysfunction showed the most notable improvement, decreasing from 1 h to 0 h. While the changes were not dramatic, they suggest a trend towards better sleep quality following treatment.

Frailty was assessed using the Fried Frailty Criteria. Before NYT treatment, the patient scored positively on four out of five criteria: exhaustion (1), weakness (1), slowness (2), and Reduced Physical Activity (1), with Unintentional Weight Loss scoring 0. The initial assessment classified the patient as frail. After NYT treatment, significant improvements were observed: Exhaustion, Weakness, and Reduced Physical Activity scores decreased to 0, whereas slowness improved from 2 to 1. Unintentional Weight Loss remained at 0. These changes indicated a shift from frail to pre-frail status, demonstrating a notable improvement in the patient's overall frailty condition.

Table 6 presents the OABSS components before and after the treatment.　The OABSS results demonstrated significant improvements across all components, particularly in the frequency of sudden urges to urinate and urge incontinence. OAB symptoms showed marked improvement, with daytime urinary frequency decreasing from 12 to eight times/day, and nocturia reduced from five to three times/night. The patient was able to engage in pelvic floor muscle exercises. 

**Table 6:** Overactive bladder symptom score (OABSS) before and after NYT administration OABSS: Overactive Bladder Symptom Score; NYT: Ninjin'yoeito. Scores for individual symptoms range as follows: Daytime Urinary Frequency (0-2), Nocturia (0-3), Sudden Strong Urge to Urinate (0-5), and Urgency Incontinence (0-5). Total OABSS ranges from 0 to 15. Severity classification: Mild (0-5), Moderate (6-11), Severe (12-15). Higher scores indicate more severe OAB symptoms.

OABSS	Before Treatment	After Treatment
Daytime Urinary Frequency	2	1
Nocturia	3	2
Sudden Strong Urge to Urinate	4	1
Urgency Incontinence	4	1

Table 7 shows the varying effects of NYT administration in two elderly female patients, demonstrating that while both cases showed improvements, the degree and nature of these improvements differed between individuals. Both cases exhibited improvements in grip strength, total testosterone levels and OABSS; however, Case 1 showed stable MMSE scores with significant improvement in the PSQI Global Score, while Case 2 demonstrated a notable increase in the MMSE score but less improvement in the PSQI Global Score.

**Table 7:** Summary of key metrics before and after NYT administration in two elderly women with frailty OABSS: Overactive Bladder Symptom Score, MMSE: Mini-Mental State Examination, PSQI: Pittsburgh Sleep Quality Index, NYT: Ninjin'yoeito, SF-36: Short-Form 36 Health Survey.

Metric	Case 1 (84-years-old) Before NYT	Case 1 (84-years-old) After NYT	Case 2 (80-years-old) Before NYT	Case 2 (80-years-old) After NYT
Grip Strength (kg)	16	18	12	18
Total Testosterone (ng/mL)	14	28	20	38
MMSE Score	28	28	12	23
Daytime Urinary Frequency (times/day)	9	6	12	8
Nocturia (times/night)	3	1-2	5	3
OABSS Total Score	12	9	13	7
SF-36 Score (Physical Functioning)	10	50	10	50
PSQI Global Score	15	8	29	24
Frailty Status	Frailty	Pre-Frailty	Frailty	Pre-Frailty

## Discussion

This case report presents compelling evidence for the potential effectiveness of NYT in managing frailty and OAB in elderly women. These two cases demonstrate varying responses to NYT administration, highlighting the improvements observed in physical strength, cognitive function, urinary symptoms, and overall quality of life, albeit to different degrees in each patient. The summarized key metrics before and after NYT administration in both cases illustrate these individual differences. Modern medicine has entered the era of precision medicine, which requires optimal treatment methods for individual patients [12,13].

Studies on the effects of NYT have been widely conducted. For instance, NYT has been shown to be effective in improving cisplatin-induced anorexia and altering the levels of peptide YY and ghrelin [14]. Additionally, NYT has been reported to improve spatial memory impairment and influence synaptic plasticity in the prefrontal cortex in a rat model of cerebral ischemia and beta-amyloid injection [15]. Furthermore, in a senescence-accelerated mouse model, NYT was shown to prevent the onset of depression-like behavior and reduce hippocampal iNOS expression [16]. These studies suggest that NYT may contribute to the improvement of various symptoms in elderly patients, and similar to the cases presented in this report, its effectiveness in managing frailty and OAB is also anticipated.

Exploring optimal treatment methods for frail patients is extremely important, and recent research using causal inference has provided new insights into the mechanism of NYT's effects [1]. That study, using non-randomized observational data from a sample of 985 women aged 65-90 years, demonstrated that NYT significantly improved OABSS and frailty scores. By applying advanced statistical techniques, such as inverse probability of treatment weighting, instrumental variables, and difference-in-differences (DiD) analyses, the study successfully illustrated the efficacy of NYT in a clinical setting. The results indicated that NYT treatment led to an average improvement of 0.8671 points in the OABSS and 0.1339 points in the frailty scores, surpassing the outcomes observed in the non-treatment group (p<0.05). Difference in differences (DiD) analysis showed that the NYT group had a significant average improvement of 0.5457 points compared to the control group. These findings highlight the practical application of causal inference techniques to reveal the effects of NYT, bridging the gap between traditional research and clinical practice. This study emphasizes the potential of NYT to exert its effects through mediating factors, such as increased testosterone levels, which may contribute to improvements in muscle strength, cognitive function, and urinary symptoms in frail elderly patients. The use of diverse statistical techniques underscores the robustness of the findings and offers a valid alternative to randomized controlled trials for exploring treatment effects in smaller clinical populations.

The increase in total testosterone levels observed in this case report (from 14 ng/mL to 28 ng/mL in Case 1 and from 20 ng/mL to 38 ng/mL in Case 2) suggests that this mediating factor may be testosterone. Previous research has shown that women with low serum testosterone levels have a higher risk of OAB, suggesting that the increase in testosterone levels after NYT treatment may contribute to the improvement of OAB symptoms [17]. Additionally, a recent study found a significant negative association between total testosterone levels and both stress urinary incontinence (SUI) and urgency urinary incontinence among frail elderly women, highlighting the role of testosterone in managing urinary symptoms [18]. This hypothesis offers an important perspective for understanding the mechanism of action of NYT, as testosterone has also been linked to improvements in lower urinary tract symptoms and nocturia [19]. NYT's influence on testosterone levels may be linked to its modulation of CYP3A enzyme activity, crucial for testosterone metabolism. CYP3A enzymes, such as CYP3A4, CYP3A5, and CYP3A7, participate in testosterone hydroxylation, a key metabolic pathway [20-22]. NYT's inhibitory and inductive effects on CYP3A in vitro suggest it may impact testosterone levels through these pathways [23]. Additionally, hormonal regulation of CYP3A expression by glucocorticoids and growth hormone further supports the link between NYT and testosterone regulation, potentially explaining the observed improvements in OAB symptoms [24].

In both cases, NYT administration resulted in significant improvement in grip strength. The 84-year-old patient's grip strength increased from 16 kg to 18 kg, whereas the 80-year-old patient showed an even more marked improvement from 12 kg to 18 kg. These improvements may also be related to the increase in testosterone levels, which is known to play an important role in maintaining and improving muscle strength. Previous research has shown that circulating testosterone levels are associated with muscle strength, including grip strength, suggesting that testosterone may contribute to improvements observed after NYT treatment [25]. Additionally, studies on NYT have indicated its potential to enhance grip strength and muscle quality in older adults with frailty, supporting its role in managing sarcopenia and improving physical function [26].

Management of OAB symptoms in frail elderly patients is particularly challenging because of the high risk of side effects from conventional pharmacological treatments [6]. Additionally, previous research has revealed that frailty is an important predictor of high post-void residual after onabotulinumtoxin A injection treatment in elderly women with severe OAB [9]. In this context, NYT may offer a viable alternative for effectively managing OAB symptoms while avoiding the common side effects associated with conventional OAB drugs, such as dry mouth, constipation, and cognitive impairment, as well as the risks of botulinum toxin treatment. Notably, studies have shown that NYT is associated with minimal adverse effects even among elderly patients, making it a safer option for this vulnerable population [27,28].

Moreover, NYT has demonstrated the ability to address multiple geriatric issues including frailty, OAB symptoms, cognitive function, and sleep quality, highlighting its potential as a comprehensive treatment option for elderly patients. The significant improvement in cognitive function observed in the 80-year-old patient (MMSE score increasing from 12 to 23) may also be related to an increase in testosterone levels, as testosterone is known to influence cognitive function and sleep [29,30]. Components like Ginseng and Reishi in NYT may further support testosterone regulation through their effects on energy metabolism and immune modulation, adding another layer of potential benefit in treating conditions associated with testosterone deficiency [31,32]. NYT has been shown to enhance hippocampal neurogenesis in a depression model, potentially explaining the cognitive improvements observed in our cases [33].

NYT is a relatively new addition to the market, but a closely related Kampo medicine, Hochuekkito (HET), has been used traditionally in Japan for centuries. HET shares a similar composition with NYT, and has been extensively studied at the molecular level. Both HET and NYT contain key herbs such as ginseng (Ren Shen) and astragalus (Huang Qi), which are commonly used in traditional Japanese medicine for the treatment of fatigue and weakness, and to enhance immune function.

HET has demonstrated anti-aging effects and cognitive improvement through mechanisms involving the modulation of monoamines such as dopamine and noradrenaline, which are critical for cognitive processes [34-36]. These similarities suggest that NYT might exert its effects through comparable pathways, further underscoring its potential as a holistic treatment strategy that mitigates the risks associated with conventional therapies and providing a safer and more effective approach for managing complex geriatric syndromes.

The observed differences in parameters between the two case reports, where each patient demonstrated slightly varied responses to NYT, indicate that individual factors may influence treatment outcomes. This variability suggests that patient-specific characteristics may affect the efficacy of NYT, which aligns with the principles of precision medicine. Further research is warranted to explore these individual differences in treatment responses, with the goal of optimizing NYT therapy for frail elderly patients. By understanding these variations, future studies can help tailor NYT interventions more precisely, potentially enhancing their effectiveness as a personalized treatment strategy. 

From the perspective of precision medicine, the multidimensional improvements observed in these cases suggest that NYT could become an individualized treatment approach for elderly patients with concurrent frailty and OAB. Precision medicine, which emphasizes a Personalized, Predictive, Preventive, and Participatory approach, has shown promise in managing conditions such as sarcopenia and cognitive decline, which are common in frail elderly populations [37]. The application of precision medicine in dementia care has demonstrated its potential in addressing the complexities of cognitive impairment through individualized treatment strategies [38]. Additionally, the need for personalized diagnostic and preventive strategies is critical for addressing sarcopenia, a key component of frailty in older adults [39]. By applying decision-making tools using discrete mathematics developed in SUI research [13,40], it may be possible to determine the indications and predict the effects of NYT treatment more precisely, aligning with the principles of precision medicine to provide safer and more effective management of complex geriatric syndromes.

## Conclusions

This case report presents two women with frailty and overactive bladder who showed significant improvements in grip strength, physical function, overactive bladder symptoms, and overall quality of life after treatment with Ninjin'yoeito. In both cases, increases in total testosterone levels were observed, along with improvements in cognitive function and sleep quality. These findings suggest that Ninjin'yoeito may offer a comprehensive approach to managing frailty and overactive bladder in patients with these conditions, potentially mediated through increased testosterone levels, and underscore the importance of further research into its mechanism of action within the framework of precision medicine.
